# Proceedings of the Sixth Deep Brain Stimulation Think Tank Modulation of Brain Networks and Application of Advanced Neuroimaging, Neurophysiology, and Optogenetics

**DOI:** 10.3389/fnins.2019.00936

**Published:** 2019-09-12

**Authors:** Adolfo Ramirez-Zamora, James Giordano, Edward S. Boyden, Viviana Gradinaru, Aysegul Gunduz, Philip A. Starr, Sameer A. Sheth, Cameron C. McIntyre, Michael D. Fox, Jerrold Vitek, Vinata Vedam-Mai, Umer Akbar, Leonardo Almeida, Helen M. Bronte-Stewart, Helen S. Mayberg, Nader Pouratian, Aryn H. Gittis, Annabelle C. Singer, Meaghan C. Creed, Gabriel Lazaro-Munoz, Mark Richardson, Marvin A. Rossi, Leopoldo Cendejas-Zaragoza, Pierre-Francois D’Haese, Winston Chiong, Ro’ee Gilron, Howard Chizeck, Andrew Ko, Kenneth B. Baker, Joost Wagenaar, Noam Harel, Wissam Deeb, Kelly D. Foote, Michael S. Okun

**Affiliations:** ^1^Department of Neurology, Fixel Institute for Neurological Diseases, University of Florida, Gainesville, FL, United States; ^2^Neuroethics Studies Program, Department of Neurology and Department of Biochemistry, Georgetown University Medical Center, Washington, DC, United States; ^3^Media Laboratory, Department of Biological Engineering, Massachusetts Institute of Technology, Cambridge, MA, United States; ^4^Center for Neurobiological Engineering, Massachusetts Institute of Technology, Cambridge, MA, United States; ^5^Department of Brain and Cognitive Sciences, Massachusetts Institute of Technology, Cambridge, MA, United States; ^6^Division of Biology and Biological Engineering, California Institute of Technology, Pasadena, CA, United States; ^7^Department of Neuroscience and Department of Biomedical Engineering and Department of Neurology, Fixel Institute for Neurological Diseases, University of Florida, Gainesville, FL, United States; ^8^Graduate Program in Neuroscience, Department of Neurological Surgery, Kavli Institute for Fundamental Neuroscience, University of California, San Francisco, San Francisco, CA, United States; ^9^Department of Neurological Surgery, Baylor College of Medicine, Houston, TX, United States; ^10^Department of Biomedical Engineering, Case Western Reserve University, Cleveland, OH, United States; ^11^Beth Israel Deaconess Medical Center, Harvard Medical School, Boston, MA, United States; ^12^Department of Neurology, University of Minnesota, Minneapolis, MN, United States; ^13^Department of Neurosurgery, Fixel Institute for Neurological Diseases, University of Florida, Gainesville, FL, United States; ^14^Center for Neurorestoration and Neurotechnology, Rehabilitation R&D Service, Veterans Affairs Medical Center, Brown Institute for Brain Science, Brown University, Providence, RI, United States; ^15^Department of Neurology and Department of Neurological Sciences and Department of Neurosurgery, Stanford University, Stanford, CA, United States; ^16^Department of Neurology and Department of Neurosurgery, Icahn School of Medicine at Mount Sinai, New York, NY, United States; ^17^Department of Neurosurgery, David Geffen School of Medicine at UCLA, Los Angeles, CA, United States; ^18^Biological Sciences and Center for Neural Basis of Cognition, Carnegie Mellon University, Pittsburgh, PA, United States; ^19^Coulter Department of Biomedical Engineering, Georgia Institute of Technology, Emory University School of Medicine, Atlanta, GA, United States; ^20^Department of Pharmacology, University of Maryland School of Medicine, Baltimore, MD, United States; ^21^Center for Medical Ethics and Health Policy, Baylor College of Medicine, Houston, TX, United States; ^22^Center for the Neural Basis of Cognition, University of Pittsburgh School of Medicine, Pittsburgh, PA, United States; ^23^Department of Diagnostic Radiology and Nuclear Medicine, Rush University Medical Center, Chicago, IL, United States; ^24^Department of Biomedical Engineering, Illinois Institute of Technology, Chicago, IL, United States; ^25^Electrical Engineering, Vanderbilt University, Nashville, TN, United States; ^26^Department of Neurology, University of California, San Francisco, San Francisco, CA, United States; ^27^Graduate Program in Neuroscience, Department of Electrical Engineering, University of Washington, Seattle, WA, United States; ^28^Department of Neurological Surgery, University of Washington, Seattle, WA, United States; ^29^Movement Disorders Program, Cleveland Clinic Foundation, Cleveland, OH, United States; ^30^Department of Neurology, Center for Neuroengineering and Therapeutics, University of Pennsylvania, Philadelphia, PA, United States; ^31^Center for Magnetic Resonance Research, University of Minnesota, Minneapolis, MN, United States

**Keywords:** deep brain stimulation, neuromodulation, epilepsy, Parkinson’s disease, tremor, optogenetics, Tourette syndrome, temporal dispersion

## Abstract

The annual deep brain stimulation (DBS) Think Tank aims to create an opportunity for a multidisciplinary discussion in the field of neuromodulation to examine developments, opportunities and challenges in the field. The proceedings of the Sixth Annual Think Tank recapitulate progress in applications of neurotechnology, neurophysiology, and emerging techniques for the treatment of a range of psychiatric and neurological conditions including Parkinson’s disease, essential tremor, Tourette syndrome, epilepsy, cognitive disorders, and addiction. Each section of this overview provides insight about the understanding of neuromodulation for specific disease and discusses current challenges and future directions. This year’s report addresses key issues in implementing advanced neurophysiological techniques, evolving use of novel modulation techniques to deliver DBS, ans improved neuroimaging techniques. The proceedings also offer insights into the new era of brain network neuromodulation and connectomic DBS to define and target dysfunctional brain networks. The proceedings also focused on innovations in applications and understanding of adaptive DBS (closed-loop systems), the use and applications of optogenetics in the field of neurostimulation and the need to develop databases for DBS indications. Finally, updates on neuroethical, legal, social, and policy issues relevant to DBS research are discussed.

## Introduction

Neuromodulation of brain structures and functions is an evolving field. Ongoing scientific and technological advancements have facilitated an improved understanding of brain networks and the neural signals involved in the signs and symptoms of a number of neuropsychiatric conditions. Novel methods of electrical current delivery have been recently applied to existing neuromodulation techniques in order to improve the understanding and the ability to more precisely affect mechanisms, function and to influence cortical and subcortical structures. The deep brain stimulation (DBS) Think Tank is an annual forum that facilitates discussion and debate about the latest scientific, technological, ethico-legal issues, social innovations, challenges, and opportunities in the field. The Sixth Annual DBS Think Tank was held in Atlanta, GA, United States from 6 to 8 May, 2018. The meeting focused on the use of novel modulation techniques and emerging areas of scientific, technological, ethical, and policy development. Specifically, the meeting addressed issues and possibilities of modulating different neuronal networks; expanding capabilities of responsive (closed-loop) DBS systems; and the therapeutic potential of targeted brain network modulation. Particular emphasis was placed upon advances and gaps in knowledge of and capabilities to affect brain electrophysiology, interface optogenetics and DBS, and on the multiple (technical, ethical, policy, and social) factors that can limit and/or de-limit these domains. We divided current proceedings in seven separate sections discussing advances in the field as follows: connectomic and network neuromodulation, advances in neurophysiological signals for DBS, new neuromodulation techniques, applications of optogenetic techniques in DBS, databases for DBS, and neuroethical, Legal and social issues in DBS.

### Brain Network Neuromodulation and Connectomic DBS

#### Leveraging Human Brain Connectomics to Improve DBS

Different stereotactic techniques are commonly used to assure proper localization of subthalamic (STN-DBS) leads in Parkinson’s disease (PD), including anatomical neuroimaging and indirect stereotactic methods. However, therapeutic benefits are likely the result of engagement and modulation of other brain regions that are interactive with specific stimulation sites and networks (Henderson, 2012; Fox et al., 2014). An improved understating of brain node and network connectivity could therefore be useful and of value to predicting and to optimizing DBS responses and outcomes (Horn et al., 2017). Using diffusion tractography, white matter tracts near the DBS electrode can be accurately identified (Coenen et al., 2011; Pouratian et al., 2011; Riva-Posse et al., 2014) and functional connectivity – a measure of the correlation in spontaneous activity – can be used to link subcortical DBS sites to effects in cortical regions (Anderson et al., 2011; Fox et al., 2014).

Recent works highlight the importance of modulating the hyperdirect pathway (connecting the STN to cortex) in the effectiveness of STN-DBS (Gradinaru et al., 2009; Accolla et al., 2016). Utilizing high-quality connectome datasets [diffusion tractography and functional connectivity from normal subjects (*n* = 1,030) and PD patients (*n* = 90)] Horn et al. (2017) were able to compute connectivity profiles of beneficial STN DBS for PD. There is a distinct pattern of connectivity with STN DBS electrodes, which directly correlated with clinical outcome. Importantly, structural and functional connectivity independently predicted DBS response.

Similarly, this technique can be used to explore or refine DBS targets and theoretically to avoid or reduce side effects of neuromodulation (Calabrese, 2016). Cognitive side effects are possible after STN-DBS. Witt et al. (2013) assessed the influence of cortical lead entry point, electrode path and position of stimulating electrode contacts on neuropsychological changes after surgery in patients with mild cognitive and semantic fluency decline. When trajectories intersected with the caudate nuclei, there was an increased risk of decline in cognition and decrements in working memory. However, these results need to be corroborated with additional larger and prospective studies. Additionally, subjects who showed a decline in semantic verbal fluency had the active electrode located outside the dorsolateral stimulation STN, and connectivity profiles showed clear differences between patients.

This technique can also be used in DBS treatment of other conditions. For example, chronic-progressive gait ataxia in patients with essential tremor (ET) can be reversed following prolonged DBS washout, and is likely due to a stimulation-induced vestibulo-cerebellar network dysfunction (Reich et al., 2016). Using volume of tissue activated (VTA) modeling, it was shown that stimulation of the more posteromedial and caudal zones of the thalamus might account for this side effect, and thus, avoiding a caudal and ventral placement might prevent such chronic side effects. Further validation of these datasets and findings is needed so that individualized DBS targets can be evermore precisely estimated using network assessments to minimize side effects.

#### Connectivity Underlies Antidepressant Response to Subcallosal Cingulate DBS

**FIGURE 1:**
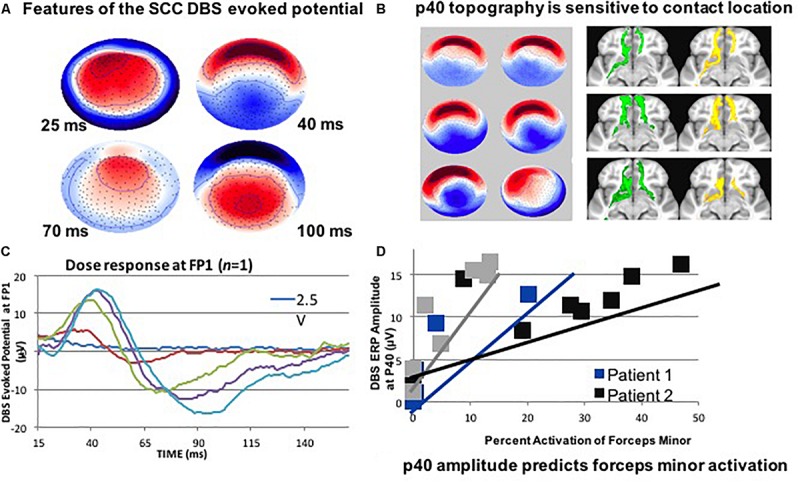
**(A)** SCC DBS evoked potentials in patients with treatment resistant depression may also be useful. It is less important to hit the visible target; it is more about hitting the correct portion of the connectome. **(B)** Use DTI: to target structures intraoperatively. **(C)** Record LFP at the DTI-optimized target. **(D)** Use high- density EEG: to detect evoked cortical responses for stimulation. This figure adapted from Cho et al. (2017).

Deep brain stimulation of the subcallosal cingulate (SCC) has recently shown promise for the treatment of therapy-resistant depression (Mayberg et al., 2005; Holtzheimer et al., 2012; Puigdemont et al., 2012), acting via modulation of specific pathological circuits. However, one of the remaining challenges is a lack of biomarkers and feedback to enable confirmation of the intended brain target. Therefore, attempts to develop a biometric of signal propagation from a novel white matter target in the SCC region are ongoing by exploiting stimulation evoked potentials as a biomarker of effective connectivity. In a recent study, four subjects were implanted in the SCC with the aid of StimVision (Noecker et al., 2018). Electroencephalographic (EEG) recordings were performed with DBS that was directed to the SCC white matter region using 2 Hz settings at 6 V. The stimulation pulse width was 90 microseconds (μs) and current was placed in a monopolar configuration. Therapeutic stimulation of 130 Hz; 3–5 V; 90 μs was resumed after the recordings were collected. A SCC DBS evoked potential (p40) was consistently elicited and detected over a period of 14 months (at four time points) (Figure 1). These results indicated that it was feasible to obtain feedback on cortical responsivity by employing a stimulation evoked potential, which can be used as a signal of optimal targeting and test–retest reliability over time. This study indicated that the p40 feature may be an activation of the forceps minor, which was consistent with previous data that linked stimulation evoked response to white matter activation (Waters et al., 2018).

#### Targeting Identified Brain Connections With DBS

As previously stated, it is becoming increasingly evident that the benefits of DBS for disorders such as ET and PD depend on the connectivity of the site of stimulation with other brain networks and regions (Horn et al., 2017; Malekmohammadi et al., 2018b). Insights into the connectivity of effective therapeutic stimulation (in comparison to ineffective stimulation) will likely enhance the ability to target deep brain structures. This could also improve the ability to develop new DBS surgical methods (such as asleep image-guided implantation, although this procedure has been performed successfully without connectivity data in many centers for years), technologies (that more precisely produce network-based neuromodulation), and therapeutic indications (such as treatment-resistant depression and other neuropsychiatric disorders including addiction; as well as certain forms of intractable pain).

Methods for evaluating connectivity include both anatomical (diffusion tensor imaging and tractography) and functional techniques [EEG, functional MRI, and invasive neurophysiological techniques including microelectrode recordings, local field potentials (LFPs), and electrocorticography]. One of the earliest demonstrations of such DBS targeting was the application of connectivity-based thalamic segmentation, which revealed discrete thalamic regions with distinctive connectivity patterns with cortical regions (Pouratian et al., 2011). Subsequent reports have elaborated on the value of tractography for thalamic targeting, using methods to both directly target the region-of-interest in the thalamus (Sasada et al., 2017; Tsolaki et al., 2018) as well as for indirect targeting, in which adjacent tracts which should be avoided are delineated (Sammartino et al., 2016). An alternate method would entail mapping the pyramidal tracts and medial lemniscus, and targeting DBS placements that are medial and anterior to these tracts, respectively (Sammartino et al., 2016). The most efficacious DBS contact for tremor control is localized within the thalamic region connected to the premotor, rather than the primary motor cortex as would be predicted by traditional, preoperative, indirect targeting methods. In ET patients, diffusion tensor imaging (DTI) based fiber tractography can aid in determining the optimal target(s) to maximally achieve tremor suppression and to reduce the number of adverse events by avoiding stimulation or lesioning of other nearby tracts, respectively (Sasada et al., 2017). Analyzing four fiber tracts important for motor control, tractography revealed a strong role of the cerebello thalamic and pre motor cortex modulation in ET patients with thalamotomy or thalamic DBS. It was observed that those fiber afferents from the cerebellum that passed through ventral intermedius nucleus (VIM) and the area anterior to the VIM were likely connected to the pre motor or motor cortex. In another study, probabilistic tractography-guided thalamic targeting was employed to treat ET (Tsolaki et al., 2018): MR imaging and clinical outcomes following thalamotomy (MR guided- Focus ultrasound) were assessed. Thalamic connectivity to pre- and post-central gyral targets was evaluated and individual thalamic target maps were generated. Using receiver operating characteristic curves to define overlapping thalamic maps, the overlap between these thalamic target maps and the MR guided- Focus ultrasound lesion was systematically weighed with respect to clinical outcome, and the connectivity differences of best clinical outcomes were compared. These investigations demonstrated that the intersection between lesion and thalamic-connectivity mapped to motor areas, while sensory targets proved to be effective in predicting the response to therapy.

While the use of tractography-guided thalamic targeting for ET has been a powerful proof of concept, an additional practical utility lies in the potential of using such approaches to either optimize therapies that have been inconsistent to date, or to develop novel applications. For example, thalamic DBS for pain is associated with inconsistent efficacy, possibly due, in part, to the lack of optimized targeting. To gain insight to the potential value of tractography-guided thalamic DBS targeting for pain, a small retrospective analysis of five patients was performed. It was shown that the pain patients who derived benefit from thalamic DBS for their pain had DBS leads that co-localized with those thalamic regions with maximal connectivity with the post-central gyrus (Kim et al., 2016). Looking ahead, it may be important for the discipline to integrate knowledge about stimulation fields, therapeutic outcomes, and tractography in order to create clinically-weighted optimal connectivity maps with which to define optimal stimulation targets (rather than to identify and engage seed regions and targets based on predefined, potentially arbitrary, anatomic segmentations; Tsolaki et al., 2018). The idea of employing multiple methods to define target site represents something of a reversal: rather than starting with a hypothesis about the optimal network engagement, this approach allows data-driven outcome-weighted delineation of networks that may prove to be contributory to improved therapeutic outcomes.

Other studies have investigated DBS targets other than the thalamus. Investigators have mapped the hyperdirect pathway from the primary motor cortex to STN, based on the hypothesis that the optimal site of STN stimulation is the site of hyperdirect pathway input. To further explore this hypothesis, hyperdirect pathways and associated STN target maps generated by two different tractography approaches (i.e., tensor-based deterministic method, and an advanced probabilistic method) were compared (Petersen et al., 2017). Both identified connections between the ipsilateral motor cortex and STN, but defined different target regions in the STN. The probabilistic method, which is based on constrained spherical deconvolution, resulted in a reconstruction of motor cortical connections terminating in the dorsolateral STN, consistent with the optimal site of stimulation. The tensor-based method resulted in a reconstruction of fewer, and more variable connections. Hence, the probabilistic method was considered to be more consistent for STN mapping.

**FIGURE 2:**
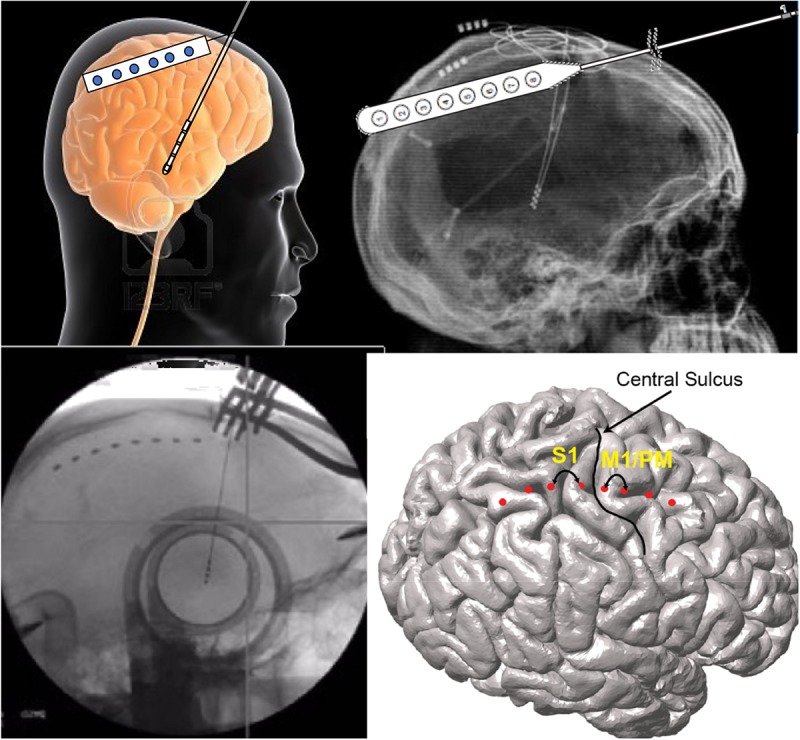
Network-based measures can be recorded during DBS surgery via the same burr hole utilized for DBS lead implantation. In these illustrations, the use of an electrocorticography strip is shown. Simultaneous signals are recorded from the motor cortices while also recording through the DBS lead.

Brain networks can also be evaluated using both non-invasive and invasive neurophysiologic methods (Figure 2). There have been several reports of either measuring biosignals in distinct nodes throughout a targeted network, or of evaluation of network-based biosignals. An example of the former is the detection of increased phase-amplitude coupling in the precentral gyrus of patients with PD, which is suppressed with therapeutic stimulation of both the STN (De Hemptinne et al., 2015) and the GPi (Malekmohammadi et al., 2018a). Another approach is to measure network connectivity by evaluating the coupling of signals in and across different nodes and to demonstrate modulation of these network-wide signals. Using such methods, two groups have recently demonstrated suppression of pallidocortical beta coherence with effective pallidal DBS and changes in cortico-subcortical functional connectivity were shown to be spatially exclusive to the motor cortex (Malekmohammadi et al., 2018b; Wang et al., 2018).

#### Individualized Network Interrogation and Targeted DBS

Neuropsychiatric disorders are increasingly being viewed as node and network-level brain dysfunctions. Apropos such a perspective, there is renewed enthusiasm for understanding the complex anatomical and functional bases of particular neuropsychiatric states and conditions. Extant, simplistic classifications of neuropsychiatric disorders based solely upon patient signs and symptoms have been limited, at best, and clinically ineffective and inefficient as worst, and therefore a taxonomy based on orthogonalized axes of psychological constructs and neural circuit dysfunction may be better suited (and of greater value) in affording a more rational (and testable) basis for developing new therapeutic methods and approaches, inclusive of DBS.

A number of brain networks have been implicated in depression, including those subserving the default mode, salience, and negative affect (Williams, 2017). The concept of targeting specific networks based on an individual biotype (e.g., neuropsychiatric phenotypes) remains an evolving construct, which may be advanced by the use of advanced neuroimaging to obtain neuroanatomical and neurophysiological information about individual patients. This may facilitate improved clinical outcomes when using more saliently targeted DBS. In an ongoing study (NCT03437928), an innovative approach to assess patients with treatment refractory depression includes the use of subacute invasive neurophysiological monitoring (i.e., stereo-electroencephalography) to gain insight to the networks involved in depressive symptomatology. The goal is to demonstrate and to confirm the capability to selectively and predictably engage distinct brain networks that are contributory to the pathologic features of depression, and to demonstrate positive therapeutic changes in such signs and symptoms through the use of network-targeted stimulation.

The study included the use of directional current steering DBS and individualized network targeting; with the aims of demonstrating that this approach to targeting was feasible and safe, and could possibly reduce depressive symptoms. Targeting networks – instead of specific structures – might prove to be crucial to individualizing therapies aimed at modulating specific brain networks important to depression (and other neuropsychopathologies). In this light, specific network modulation may also be viable and of value in treating obsessive compulsive disorder (OCD) (Banks et al., 2015). Previously, a number of targets have been used to treat OCD. Recent tractographic data show network-level pathophysiology that can be targeted with DBS. It has been reported that subdivisions of the anterior limb of the internal capsule were found to vary substantially as viable targets for DBS in treating OCD. However, some loci did prove to be consistently useful targets, with the most notable effects being produced by stimulating regions most densely connected to the orbitofrontal cortex. Future studies are aimed at continuing these investigations to (1) develop new technologies to delineate symptomatic networks in neuropsychiatric disorders and (2) to assess the effect of neuromodulation that targets different networks and regions identified via advanced neuroimaging to be participatory in depressive (and other psychiatric disorders’) signs and symptoms.

#### Network Assessment in Movement Disorders

Given mounting evidence to support the possibility of obtaining good clinical outcomes in the absence of neurophysiological mapping – and multi-dimensionally (e.g., clinically, technologically, economically); an important question is what the future of intracranial neurophysiology portends in an era of increasing use of real-time MRI-guided and CT-verified DBS implantation in asleep patients. One possibility is that network mapping, rather than mapping of a single target structure, will be important to improve the efficacy – and ultimate clinical effectiveness and value of DBS. For instance, as evidence emerges for separate networks that may be therapeutically responsive to differential stimulation (e.g., speech, gait, non-motor symptoms of PD) (Humphries and Gurney, 2012), techniques will be needed to identify the optimal locations within these networks for DBS placement. Additionally, if cortical sensors [e.g., electrocorticography (ECoG)] become an important component of closed-loop DBS systems, intraoperative functional connectivity mapping of the target network may be required for (optimized) placement of both sensing and stimulating electrodes.

**FIGURE 3:**
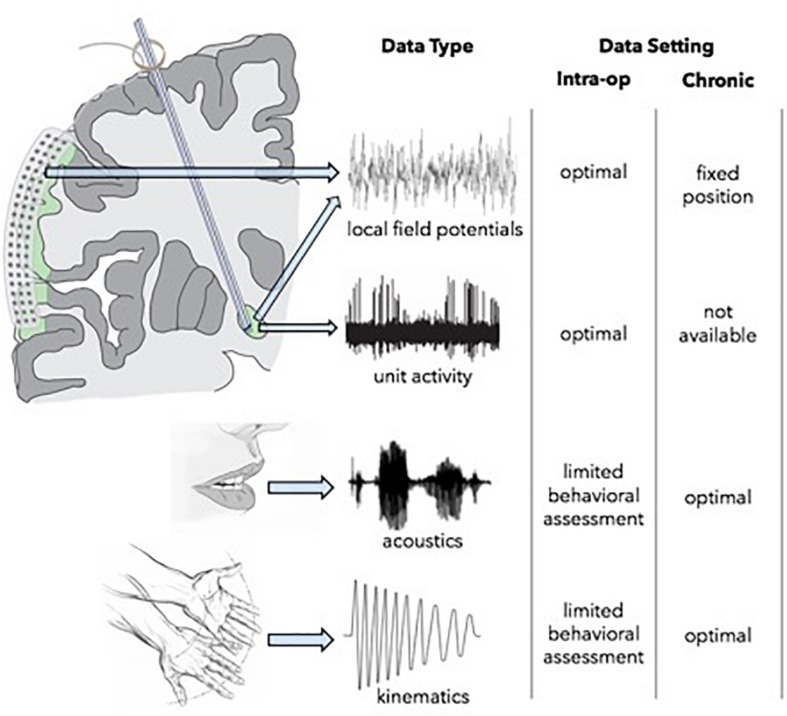
Intracranial neurophysiology opportunities for network assessment.

Opportunities for multi-scale, high-resolution neurophysiological data collection are most robust intraoperatively, but assessments are limited by available time and by the physical constraints that restrict the complexity of behavioral tasks which can be engaged (Figure 3). The use of simultaneous ECoG during DBS implantation, however, can serve as a powerful tool for identifying optimal network nodes. Using this technique, single unit and LFP recordings from the target structure, as well as LFPs from functionally connected areas of the cortex can be simultaneously obtained (Panov et al., 2017). ECog has been used to map pathological and physiological networks in PD and dystonia. Additionally, (and perhaps of greater importance), these studies have elucidated processes of information transfer between the basal ganglia and cortex during limb movement and speech production, which may afford information about which signals are best suited for closed-loop DBS systems capable of adapting to specific behavioral signs and symptoms. Once a patient is implanted, intracranial neurophysiological recording locations and modalities are fixed, but the subject can participate in much longer trials of behavioral testing, either in a subacute setting with externalized leads, or chronically following implantation of a bi-directional pulse generator. Lastly, functional imaging modalities can be used in multiple settings facilitating longitudinal assessment. For example, pre-operative MEG or fMRI data can be compared to those data obtained in subacute or chronic post-operative periods in order to measure target network engagement and other network effects of stimulation over time.

#### Parametric Subtracted Post-ictal Diffusion Tensor Imaging for Guiding Direct Neurostimulation Therapy

New techniques in tractography are being investigated as methods which can be used to identify aberrant brain networks and potential foci of epilepsy (Sivakanthan et al., 2016). Using tractography to identify a specific ictal focus, implanting devices for white matter modulation might afford benefit to those epilepsy patients for whom other treatments are not viable. Currently, the goal is to capture ictal and post-ictal states via imaging tools in order to enable more individualized therapies. The development and use of newer techniques will allow greater precision in targeting domains of the refractory epileptogenic network(s) (Nemtsas et al., 2017; Rossi, 2017). This will optimize targeting and trajectories to access the involved network beyond simply an anatomical target, to more fully and specifically modulate the propagation pathway. Ongoing work aims to utilize parametric methods to subtract post-ictal DTI to identify such potential targets for modulation (Garibay-Pulido et al., 2018). This technique has been helpful both for studying ictal states and for identifying acute and chronic changes in network activity in epilepsy patients. There are statistical differences between pre- and post-seizure activity patterns that allow the visualization of the epileptic network. From this visualization, electrodes can be modeled into the DTI technique to reveal probabilistic stimulation-induced network activations. To be sure, these developments are promising, and additional steps and validation, including fiber “cable modeling” via the use of Hodgkin-Huxley equations, will be necessary and important to validating and refining these methods.

### Recent Advances in Neurophysiological Signals for DBS

#### STN Recordings to Inform Closed Loop DBS in Parkinson’s Disease

Resting state LFP neural recordings from the PD STN in intra- or peri-operative studies have demonstrated exaggerated oscillatory neuronal activity and synchrony in both the alpha (8–12 Hz) and beta (13–30 Hz) bands (Maling et al., 2018). The extent of attenuation of resting state beta band power during therapeutic doses of both dopaminergic medication and high frequency (HF) DBS has been correlated with improvement in bradykinesia and rigidity. This suggests that STN resting state beta band power is a physiological marker of PD and could be a potential control variable for closed loop or adaptive (a)DBS. The resting state beta band is conserved over time and across different resting postures (Bronte-Stewart et al., 2009); it is similar between STN nuclei; of greater power in more affected STN (De Solages et al., 2010); it is a property of the functionally connected Parkinsonian sensorimotor network; and its phase is coupled with gamma band amplitude in the STN and M1 cortex (Whitmer et al., 2012). It is attenuated during HF DBS and after washout of chronic STN DBS (Whitmer et al., 2012; Trager et al., 2016); it is attenuated during tremor, but it is not highly correlated to PD motor signs (Shreve et al., 2017). Taken together, these findings prompt the question of whether resting state beta band power is a validly good control variable for aDBS in freely moving patients.

**FIGURE 4:**
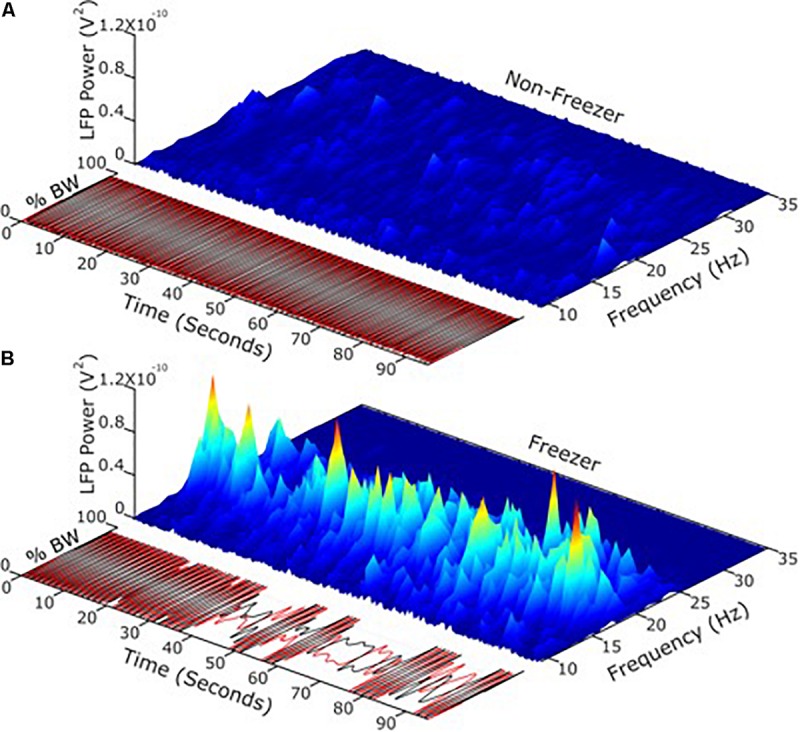
**(A)** Using LFP’s and neurophysiology to define and address freezing of gait in Parkinson’s disease. **(B)** Temporal fluctuations and the extent of changeable behavior of STN band neural activity during movement differentiated PD freezers from non-freezers.

Apropos this question, it is now possible to record synchronized neural and kinematic signals from an implanted sensing neurostimulator and from wearable sensors respectively, in freely moving human subjects (Activa^®^ PC + S, Medtronic, Inc., FDA IDE approved). It has been demonstrated that the transition from the resting state to a moving state (such as walking) is associated with a change in the LFP spectrum, which was more prominent in the akinetic rigid versus the tremor dominant PD phenotypes (Quinn et al., 2015), and in PD freezers as compared to non-freezers (Syrkin-Nikolau et al., 2017). Beta band power during movement was attenuated in a voltage dependent pattern during randomized presentations of differing voltages of STN DBS (Whitmer et al., 2012). Different beta sub-bands showed distinct sensitivities to attenuation during STN DBS. Based upon this, it was investigated whether the more or less sensitive sub-band was superior as a control variable for aDBS (Afzal et al., under review). Temporal fluctuations and the extent of unpredictable behavior of STN band neural activity during movement (i.e., movement band) differentiated PD freezers from non-freezers (Figure 4). Movement band burst durations and entropy (i.e., sample entropy) were longer and greater respectively in freezers compared to non-freezers during gait without FOG, and were longer/greater in freezers compared to non-freezers and during periods of FOG (Syrkin-Nikolau et al., 2017). Temporal properties of the resting state beta band did not differ between freezers and non-freezers. Longer movement band burst durations and impaired gait improved during 60 and 140 Hz STN DBS in freezers, but short burst durations and normal gait in non-freezers were unchanged. These results suggest that gait impairment and FOG are characterized both by more prolonged periods of pathological neural synchrony and by unpredictability or chaos in STN beta rhythms, which disrupts normal sensorimotor processing. The effect of STN DBS was directed toward mitigating pathological neural activity related to gait, and toward restoring greater physiological information processing in the sensorimotor network, so as to improve gait and FOG in PD freezers. This information affords new neural control variables that may be more relevant in freely moving PD subjects than the resting state beta band.

#### Pallidal and Cortical Recordings in PD: Prospects for Pallidal Closed Loop DBS

There is increasing use of closed-loop DBS in PD, with most groups utilizing STN signals as the neurophysiological marker to enable aDBS. However, the globus pallidus interna (GPi) is also an important locus in the pathological network of PD, and is commonly targeted for the treatment of refractory motor symptoms (Ramirez-Zamora and Ostrem, 2018). Investigating potential pallidal and cortical signals might allow for reduction of adverse side effects. Cortical signals have greater signal to noise ratio than signals recorded from deep subcortical structures (De Hemptinne et al., 2015). However, recent work has been focusing on developing and characterizing pallidal interval recordings as potential markers that can be studied in advanced, adaptable DBS to improve tremor management in PD, reduce adverse effects (including brittle responses, tolerance), reduce current drain, and incorporate variability related to circadian rhythms (Swann et al., 2018). Prior work has validated exaggerated oscillatory neuronal activity and synchrony in the beta bands as a biomarker for aDBS.

Thus, current studies aim to improve the closed loop algorithm and to analyze smaller windows of data to test different refresh rates. Detector sophistication will be critical to acquiring and assessing signals, and several critical questions remain regarding whether to employ single threshold or center frequency; the ideal rate of signal updates; ramp and onset times, and the potential for energy savings, technical sources of variance, long term benefit and signal reliability and reproducibility afforded by this technique. Moreover, the increasing complexity of these signals will likely require computerized or algorithmic programing to mitigate long programing visits, and this prompts questions and concerns about capability and use of bio-informatic systems to facilitate these approaches (see also above, re: bio-informatic systems development, progress and challenges).

#### Adaptive Pedunculopontine Nucleus (PPN) DBS for Treatment of Freezing of Gait

FOG affects approximately 78% of PD patients and is a major cause of falls, and determinant of quality of life. Animal research suggests that the GPi and PPN contribute to the FOG in advanced PD (Thevathasan et al., 2018). Despite several clinical trials reporting improvement in PD motor symptoms with DBS, treatment of FOG remains challenging. PPN and PPN + STN DBS trials have been attempted for management of refractory FOG, and have yielded mixed and inconclusive findings (Thevathasan et al., 2018). A recent study (NCT02318927) evaluated the feasibility and potential effectiveness of closed-loop PPN for medication-refractory FOG in patients with PD. Five (5) subjects with on-medication FOG were implanted with bilateral open-loop GPi DBS to address the cardinal PD motor symptoms. In addition, bilateral PPN DBS was used as an adaptive closed loop strategy to specifically address FOG. PPN recordings were precisely characterized and utilized for measuring neural correlates to deliver responsive stimulation. PPN low frequency power (1–8 Hz) tended to increase during gait, while GPi beta frequency tended to decrease during movement. Several challenges and complications were encountered during the study, as consistent with other studies in advanced PD patients. For example, recording signals were not consistent in all electrode contacts or across both hemispheres. But despite challenges, the study’s primary goal of improving FOG was met, albeit with some limitation: the Closed loop-DBS protocol produced a greater than 40% improvement in FOG at 6 months in a minimum of 3/5 subjects. However, adverse events including scalp erosions and infections limited the feasibility of Closed loop-DBS in all patients, and disease progression led to worsening of motor and non-motor symptoms over time.

#### Thalamic and Cortical Signals for Development of Closed Loop DBS in Essential Tremor

Closed-loop stimulation has similar efficacy to open-loop, and in some contexts, closed-loop stimulation might be better than traditional DBS (Little et al., 2016). Chronically implanted sensing/stimulating devices (i.e., Activa RC + S) provide the capability to acquire large volumes of diverse data in real-life settings. These data can be useful in developing more sophisticated, accurate and reliable biomarkers than those that are currently available and used. Yet, closed loop-DBS also fosters a number of questions that are, and will be, important to consider; for example, is too much stimulation equally as problematic as too little stimulation – and if so, in what ways? For which diseases or disease states is closed loop-DBS best suited/most effective? Can closed loop-DBS afford more specific cortical biomarkers that can inform and guide improved modulation, clinical outcomes, and reduced side/adverse effects?

The Medtronic Activa PC + S device enables the use of a cortical strip for sensing/recording and a DBS electrode for stimulation in the treatment of patients with refractory tremor. The need for tremor control fluctuates greatly during the day and theoretically, a closed loop system could minimize concerns about side effects related to over- and/or under-stimulation (Kuo et al., 2018). Investigators have employed a cortical strip placed over M1, where beta power was used to trigger stimulation in the thalamus using a dual threshold method and machine learning in a subject-specific DBS model (Houston et al., 2019). Thresholds were set to optimize and minimize under-stimulation (at the relative calculated cost of over-stimulation). Assessments of three (3) subjects performed by blinded clinicians demonstrated that there was no significant difference in effectiveness between open-loop and closed loop stimulation, although at least one clinician evaluated spiral drawing by one subject to be better with closed-loop stimulation. Using machine learning tools, ongoing studies are attempting to define optimal parameters for open-loop stimulation for a given patient. A voluntary brain computer interface control method allows patients to control stimulation for patient-driven mitigation of side effects [depending on the function(s) being performed]. At present, it remains to be seen how accurate detection of movement and/or intention by cortical strips can be used to develop additional algorithms that can shift depending on a patient’s functional state. Furthermore, exploratory assessment revealed that patients’ feelings about their relative agency and/or autonomy may differ depending upon whether they have or do not have control over the level of DBS stimulation. Preliminary findings suggest that patients’ sentiments vary widely (Gilbert, 2015).

#### Update on Brain Signals for Closed Loop DBS in Parkinson’s Disease

Although DBS treatment of PD is an active field of research, many of the initial challenges and limitations of these treatments continue to be investigated. Meta-analysis of multiple DBS studies showed great variability in lead location (Perestelo-Perez et al., 2014). About one-third of implanted electrodes are revised or removed, and about 50% of these are due to suboptimal lead location. Lead localization and placement are integral to the success of DBS treatment, and this remains a challenge (Ellis et al., 2008). In part, this reflects varied anatomical preferences for DBS electrode implantation between neurosurgical practitioners and groups.

Thus, it becomes important to ask whether we really know where the target is, and if it is the “best” target. For example, in treating PD, tremor might be more responsive when the STN is stimulated in one subregion, and bradykinesia may improve more with stimulation of another subregion. In a recent study, researchers attempted beta-triggered closed- loop DBS in primates that were engaged in an acute reaching task. It was found that greater improvements in rigidity were obtained using closed- loop DBS (vs. traditional DBS), and that rigidity improved more during certain aspects of the movement (Hendrix et al., 2018). These results fortified the fact that biomarkers are dynamic and can fluctuate depending on functional state (e.g., sleep vs. awake), and that multiple algorithms might be needed to affect various target symptoms dependent on behavioral states. Biomarker detection using machine learning control algorithms might be required and may need to be adaptive over time to compensate for neuroplasticity. Data collected from cortical arrays implanted over M1 in primates showed that STN DBS reduced cortical-subcortical coupling (Wang et al., 2017). During off-DBS, the animal took longer to return to movement, but with GPi DBS-ON, there was a pattern of re-synchronization during the rest phase and de-synchronization during movement.

### Evolving Neuromodulation Techniques

#### Temporal Interference and Deep Brain Stimulation

**FIGURE 5:**
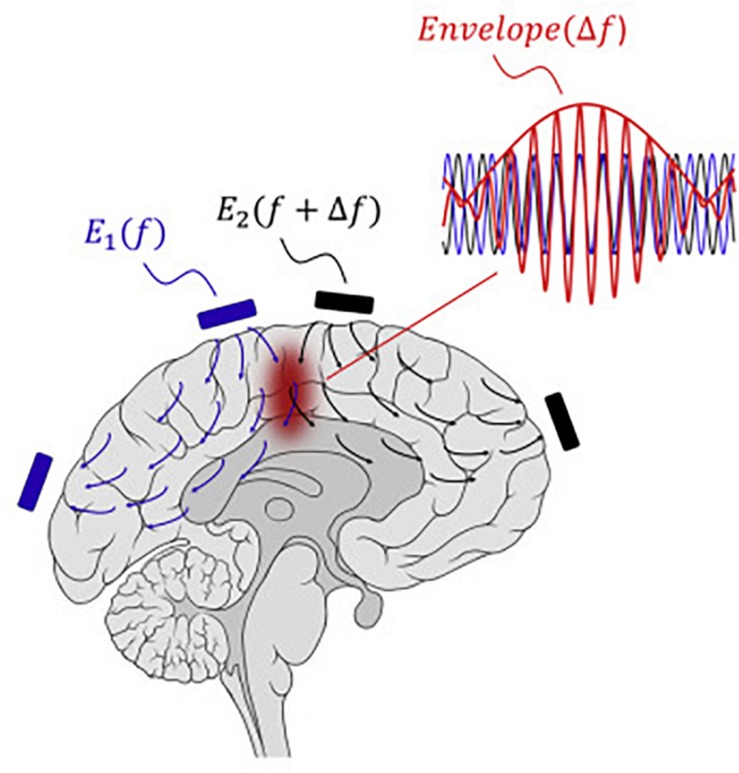
Temporal interference as a non-invasive DBS technology.

Boyden and Grossman recently developed a non-invasive approach to electrically stimulating neurons at depth (Grossman et al., 2017). By delivering multiple electric fields at frequencies too high to recruit neural firing, but which differ by a frequency within the dynamic range of neural firing, neurons could be stimulated throughout a region wherein interference between the multiple fields results in a prominent electric field envelope that is modulated at the difference frequency. This temporal interference (TI) was validated in modeling and physics experiments, and these studies verified that neurons in the living mouse brain could follow the electric field envelope (Grossman et al., 2017). The utility of TI stimulation was demonstrated *in vivo* by stimulating neurons in the mouse hippocampus without recruiting neurons of the overlying cortex. By altering the currents delivered to a set of immobile electrodes, different motor patterns in living mice could be ‘steerably’ evoked (Figure 5).

#### Update on Coordinated Reset

Increasingly, data from both preclinical models and direct recordings from patients undergoing DBS surgery support a mechanistic role for changes in the presence and dynamics of frequency-specific, synchronized oscillations across the dopamine-depleted basal ganglia thalamocortical ‘motor’ circuit (Nini et al., 1995; Brown et al., 2004; Mallet et al., 2008). Coordinated reset (CR) is an experimental neuromodulation paradigm engineered by Tass (2003) to desynchronize ‘pathological’ oscillatory activity across neuronal populations through the intermittent, pseudo-randomized delivery of brief, low-intensity, spatially-distributed pulse trains. A key advantage of CR relative to traditional DBS is that its effects should be achieved using lower individual pulse amplitudes (Tass, 2003), as the goal of the spatiotemporally-randomized stimuli is to break the otherwise hyper-synchronized target region into multiple, independent clusters. It is believed that an additional effect of this clustering phenomenon is to disrupt spike-timing-depending plasticity that may be reinforcing the persistence of the abnormal synaptic connectivity underlying the development and persistence of pathological activity. The end result being that even intermittent CR therapy may yield benefits that outlast cessation of stimulation by hours or days (Tass, 2003; Tass et al., 2012; Adamchic et al., 2014). From a therapeutic standpoint, a major benefit of CR DBS is that it may reduce the risk of provoking side-effects attributable either to the spread of electrical current outside of the target region or to chronic, continuous stimulation of the target itself (Ferraye et al., 2008; Moreau et al., 2008; Van Nuenen et al., 2008; Xie et al., 2012), and in these ways, reduce the overall duty-cycle of DBS delivery. Hence, a CR DBS approach that involves the intermittent delivery of low-intensity pulses may provide comparable motor benefit but be less disruptive to cognitive, affective, and even sensorimotor processing, while concomitantly reducing power consumption requirements.

At this time, the potential of CR stimulation to mitigate oscillatory activity is well-supported by theoretical models (Hauptmann et al., 2007; Hauptmann and Tass, 2009; Guo and Rubin, 2011; Lucken et al., 2013; Fan and Wang, 2015); however, *in vivo* preclinical or clinical evidence of its effects has been limited to three preliminary reports (Tass et al., 2012; Adamchic et al., 2014; Wang et al., 2016). On-going studies involve the use of the 1-methyl-4-phenyl-1,2,3,6-tetrahydropyridine (MPTP) non-human primate model of parkinsonism to better understand the acute, sub-acute and long-term efficacy profile of CR relative to traditional DBS, as well as to define its effects on neural activity across the basal ganglia thalamocortical motor circuit. The continuing goal is to acquire data that will facilitate the design of future human trials while further advancing an understanding of the role of synchronized oscillations in individual parkinsonian motor sign manifestation. The preliminary data acquired to date are extremely supportive of a potential role for CR in treating parkinsonian motor signs, but further point to potential interaction between its effects and baseline motor severity, lead localization, as well as the relative value of concurrent dopamine replacement therapy. To be sure, successful implementation of the CR DBS will require an effective re-dosing strategy to ensure long-term stability of its therapeutic effects. Potential options could range from open-loop strategies that involve applying CR at regimented dosing epochs, to more physiologically-dependent, demand-controlled approaches that are based on recordings derived either from the DBS lead itself or from secondary intracerebral recording arrays.

### Advancing Applications of Optogenetic Techniques to DBS

#### Deep Brain Networks and Circuits for Multiple Behavioral States: Sleep, Arousal, Reward, Locomotion, and Addiction

As reports from previous DBS Think Tanks, and a growing body of international literature reveal, DBS can be a powerful therapeutic option for intractable movement and affective disorders, with benefits often being dramatic and manifested as instantaneous improvements in function and/or pathologic presentation. However, because electrical stimulation is non-specific, the mechanisms underlying or involved in the effects of DBS remain less than fully understood, and in some cases, are a source of controversy. The application of optogenetics to DBS challenged the traditional perception that DBS in the STN acts mainly by inhibiting local cell bodies at the stimulation site. Optogenetics uses genetically encoded, light-sensitive proteins to modulate or monitor the function of specific cell types within living heterogeneous tissue. In one of the first demonstrations of the power of optogenetics, we showed that control of axons in the stimulation area was sufficient to restore motor behavior in PD models (Gradinaru et al., 2009).

**FIGURE 6:**
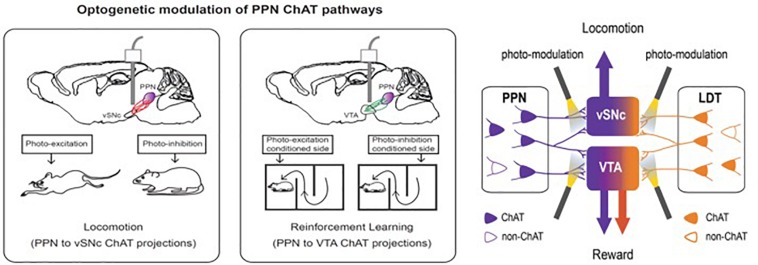
Bidirectional manipulation of cholinergic cells in the PPN exert opposing effects on locomotor behavior and reinforcement learning via specific projections to the ventral substantia nigra pars compacta (vSNc) and the ventral tegmental area (VTA). This figure adapted from Cho et al. (2017).

At least partly based upon these findings, a number of other targets for DBS are now being investigated for various indications. Examples include the neuromodulatory circuitry of two deep brain regions, the cholinergic mesopontine tegmentum [comprising the PPN and the laterodorsal tegmentum (LDT)] and the dopaminergic neurons of the dorsal raphe nucleus (DRN^DA^) (Xiao et al., 2016). The use of neurotechnology revealed details of both circuits that may ultimately lead to new therapeutic targets for movement and addiction disorders (PPN circuits), and sleep/arousal disorders (DRN^DA^ circuits) (Figure 6). Bidirectional manipulation of cholinergic cells in the PPN exerts opposing effects on locomotor behavior and reinforcement learning via specific projections to the ventral substantia nigra pars compacta (vSNc) and the ventral tegmental area (vTA). Additionally, DRN^DA^ activity increases in response to salient stimuli irrespective of valence, is correlated with sleep–wake states, and can bi-directionally modulate arousal.

##### Sleep and arousal modulation

**FIGURE 7:**
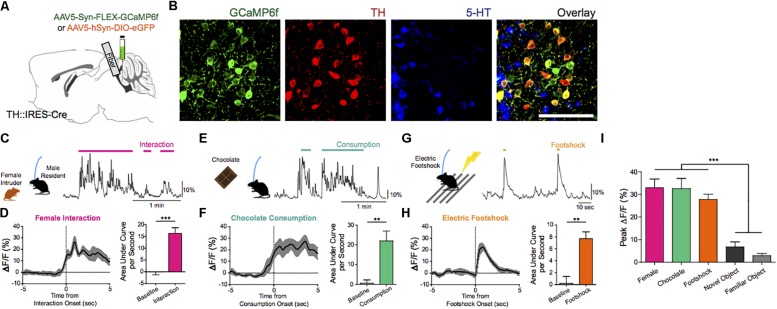
Endogenous and optogenetically driven DRN^DA^ firing. **(A)** TH-Cre mice were injected with AAV5-Syn-FLEX-GCaMP6f or AAV5-hSyn-DIO-EGFP and implanted with an optical fiber into the DRN for fiber photometry. **(B)** Confocal images of GCaMP6f+ (green) neurons show co-localization with TH+ neurons (red), but no overlap with 5-HT+ neurons (blue). Scale bar, 100 μm. **(C)** Social interaction between a male DRN^*DA-GCaMP6f*^ resident mouse and a female intruder were associated with increased DRNDA activity; the trace is a representative recording with interaction bouts indicated. **(D)** Left: female interaction caused an increase in fluorescence at the onset (first interactions only). Right: quantification of the area under the curve per second (AUC) during the interaction (0–5 s) shows that social interaction caused significant increase in DRNDA activity from baseline (−5 to 0 s) (*n* = 7 DRN^*DA-GCaMP6f*^ mice; paired *t* test, t6 = 11.97, ^∗∗∗^*p* < 0.001). **(E)** Chocolate consumption by a DRN^*DA-GCaMP6f*^ mouse increased DRNDA activity; representative recording. **(F)** Left: DRNDA activity was increased upon chocolate consumption. Right: AUC quantification during consumption (0–5 s) compared with baseline (−5 to 0 s) shows that food consumption is associated with significant fluorescence increase (*n* = 7 DRN^*DA-GCaMP6f*^ mice; paired *t* test, t6 = 4.273, ^∗∗^*p* < 0.01). **(G)** Electric footshocks (0.25 mA, 1 s) were delivered; representative DRNDA trace during two consecutive footshocks. **(H)** Left: footshock induced phasic DRN^DA^ activation. Right: DRN^DA^ activity after footshock (0–5 s) was significantly increased relative to baseline (−5 to 0 s) (*n* = 7 DRN^*DA-GCaMP6f*^ mice; paired *t* test, t6 = 5.763, ^∗∗^*p* < 0.01). **(I)** Peak DRNDA fluorescence values during female interaction, chocolate consumption, and electric footshocks were significantly higher than those during novel and familiar object interaction (*n* = 7 DRN^DA-GCaMP6f^ mice; one-way ANOVA, F_4,30_ = 22.77, *p* < 0.0001, Bonferroni post hoc analysis, ^∗∗∗^*p* < 0.001).

**FIGURE 8:**
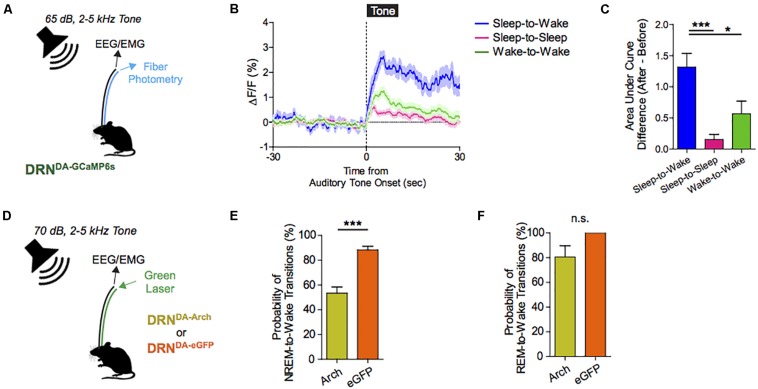
Dorsal raphe nucleus dopaminergic (DRN^DA^) activity escalates in response to salient stimuli irrespective of valence; correlates with sleep–wake states, and can bidirectionally modulate arousal. **(A)** Experimental paradigm. **(B)** Auditory cues are associated with time-locked increases in DRN^DA^ activity. **(C)** DRN^DA^ activity increase, as indexed by the difference in the area under the curve between before and after tone presentation, was larger when auditory tone induced sleep-to-wake transitions than when it was turned on while awake or when it failed to cause sleep-to-wake transitions (*n* = 7 DRN^DA–GCaMP6s^; One-way ANOVA, *F*_2,18_ = 10.79, *p* < 0.001, *Post hoc* Bonferroni analysis, ^∗∗^*p* < 0.01). **(D)** Experimental paradigm. **(E)** Time-locked DRN^DA^ inhibition decreased the probability of NREM-to-wake transitions upon auditory cues (*n* = 6 DRN^DA–Arch^, *n* = 4 DRN^DA–eGFP^; Two-tailed, unpaired *t*-test, ^∗∗∗^*p* < 0.001. **(F)** No significant change in the probability of REM-to-wake transitions (*n* = 6 DRN^DA–Arch^, *n* = 4 DRN^DA–eGFP^; Two-tailed, unpaired *t*-test, *p* > 0.1).

The ability to awaken in response to salient stimuli and to remain alert when awake, particularly upon perception of relevant environmental danger or crying offspring, holds obvious biological importance in terms of survival and fitness of self, kin, kith, and/or species. Our work using mouse models suggests that DRN^DA^ neurons play a key role in this process (Cho et al., 2017). Using simultaneous fiber photometry and polysomnography (EEG/EMG), time-delineated DRN^DA^ activity upon exposure to arousal-evoking salient cues, irrespective of hedonic valence, was observed. As well, fluctuations of DRN^DA^ activity across sleep–wake cycles were seen, with highest activity during wakefulness versus sleep states. Both endogenous and optogenetically driven DRN^DA^ firing were associated with arousal from sleep (Figures 7, 8). Conversely, chemogenetic DRN^DA^ inhibition opposed wakefulness. Finally, time-locked DRN^DA^ inhibition reduced the probability of an immediate sleep-to-wake transition upon delivery of an unconditioned tonal stimulus.

Cumulatively, these data suggest that DRN^DA^ neurons modulate arousal and can promote awakening by salient stimuli. While the ability to rouse from sleep in response to alerting stimuli is an evolutionarily conserved survival strategy, it may have negative sequelae in modern human populations: insomnia or hypersomnia triggered by malfunctioning arousal-promoting circuits is a morbid societal burden (Roth, 2007). Going forward, strategies targeting DRN^DA^ activity may have utility both in the treatment of primary sleep–wake disorders and sleep/arousal disturbances secondary to myriad neuropsychiatric diseases, including depression, bipolar disorder, and schizophrenia.

##### Modulation of reward

The mesopontine tegmentum provides major cholinergic inputs to the midbrain and regulates locomotion and reward. To delineate underlying projection-specific circuit mechanisms, we employed optogenetics to control mesopontine cholinergic neurons at the somata and at divergent projections within distinct midbrain areas. Bidirectional manipulation of cholinergic cell bodies in the PPN exerted opposing effects on locomotor behavior and reinforcement learning. Motor and reward effects were separated by limiting photostimulation to PPN cholinergic terminals in the vSNc or in vTA, respectively. LDT cholinergic neurons also form connections with vSNc and vTA neurons; however, although photo-excitation of LDT cholinergic terminals in the vTAc aused positive reinforcement, LDT-to-vSNc modulation did not alter locomotion or reward. Therefore, the selective targeting of projection-specific mesopontine cholinergic pathways may offer increased benefits in treating movement and addiction disorders.

#### Optogenetic Techniques to Facilitate Modulation of Motor Circuits

The identification of distinct cell types in the basal ganglia has been critical to an understanding of basal ganglia function and the treatment of neurological disorders. The external globus pallidus (GPe) is a key contributor to motor suppressing pathways in the basal ganglia, yet its neuronal heterogeneity has not been engaged as a resource for therapeutic interventions. Optogenetic techniques may enable cell specific identification and stimulation of GPe networks that can be modulated by DBS (Gittis et al., 2014). The indirect pathway is overactive in PD and suppression of GPe neurons using these techniques might both increase activity in GPe and improve movement. However, experiments in mouse models failed to demonstrate a clear benefit in motor symptoms. In recent studies, optogenetic interventions that dissociate the activity of two neuronal populations in the GPe, elevating the activity of parvalbumin (PV)-expressing GPe neurons over that of Lim homeobox 6 (Lhx6)-expressing GPe neurons, reinstates movement in DA-depleted mice, and decreases pathological activity of basal ganglia output neurons for hours past stimulation (Mastro et al., 2017). These results establish the utility of cell-specific interventions in the GPe to target functionally distinct pathways, with the potential to induce long-lasting recovery of movement despite the continued absence of DA.

#### The Use of Optogenetics to Understand Addiction Circuitry

**FIGURE 9:**
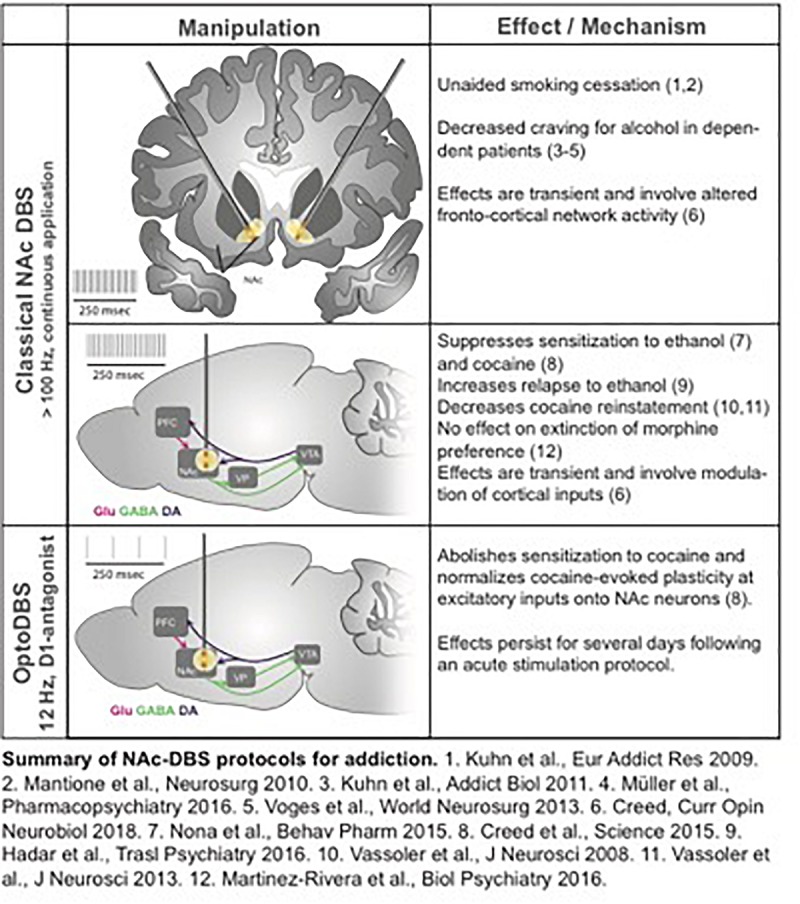
Approaches to Addiction DBS.

Addiction can be regarded – at least to some extent – as a neural circuit disorder; functional imaging has identified the cortico-accumbal-pallidal network as a locus of altered resting state connectivity in patients with addiction, and the nucleus accumbens (NAc) has been proposed as a target for DBS (Figure 9). NAc-DBS suppresses drug seeking and sensitization to drug-associated cues, although these behavioral effects are transient, and paradoxical increased substance consumption has been observed (Hadar et al., 2016; Creed, 2018). Moreover, a better understanding of the underlying mechanisms of the NAc-DBS may be useful for optimizating stimulation protocols.

Understanding mechanisms underlying altered functional connectivity in addiction could be leveraged to design targeted neuromodulation protocols to normalize circuit function and abolish behaviors associated with addiction. For example, cocaine exposure potentiates prefrontal cortical inputs to the NAc via the insertion of calcium-permeable AMPA receptors, and optogenetically driving these inputs between 10 and 15 Hz triggers a signaling cascade that reverses this synaptic potentiation and abolishes drug sensitization and seeking (Pascoli et al., 2011). Based on these observations, an optogenetically-inspired DBS (OiDBS) protocol was developed; this protocol combined 12 Hz stimulation with a D1 dopamine receptor antagonist (Creed et al., 2015). The D1R antagonist was necessary to prevent D1R-dependent signaling which inhibits de-potentiation. OiDBS normalized synaptic transmission and reduced locomotor sensitization following cocaine exposure. If translated to humans, an OiDBS protocol would have several advantages over traditional DBS protocols. It would significantly reduce stimulation time, which would extend battery life and/or could be instrumental to non-invasive stimulation methods. Stimulation protocols could also be tailored to normalize circuit function associated with diverse symptoms of addiction, such as craving vs. withdrawal-induced negative affect. To this end, neural signatures of behavioral symptoms would need to be identified. Recording from multiple sites and using altered synchrony across brain areas to trigger DBS in a closed-loop manner would represent a major therapeutic advance. Further, the use of OiDBS could extend beyond addiction to other disorders characterized by altered function of neural circuits, such as chronic pain, mood, and neurodevelopmental disorders.

#### Use of Optogenetics in Alzheimer’s Disease Circuitry

While neural stimulation has opened up a new class of therapeutics for a number of neurological and psychiatric diseases, there has been little work to examine how such stimulation could be used to treat Alzheimer’s disease (AD), the most common form of dementia. The lack of research on neural stimulation in AD likely stems from the fact that little is known about how neural activity fails in AD or how neural activity affects molecular pathology. Therefore, to determine how brain stimulation could potentially be used to treat AD, we identified deficits in neural activity in mouse models of AD and determined the effects of neural stimulation on molecular and cellular pathology (Iaccarino et al., 2016).

Because spatial navigation deficits are one of the earliest symptoms of AD and the hippocampus is one of the neuroanatomical areas first affected by the disease, we examined how hippocampal neural activity changes in AD (Fox et al., 1996; Deipolyi et al., 2007). Using a virtual reality behavior paradigm to record and manipulate neural activity in transgenic mice, the primary animal model of AD, deficits in hippocampal neural activity were revealed early in the progression of the disease. These deficits occurred in the same patterns of activity that we and others previously found to be involved in memory-guided decisions in spatial navigation tasks (Carr et al., 2012; Pfeiffer and Foster, 2013; Singer et al., 2013). Subsequently, we found that driving gamma activity, which is lacking in AD mice, mobilized the immune system to remove pathogenic proteins. Specifically, driving 40 Hz neural activity via optogenetic stimulation recruited microglia -the primary immune cells of the brain – to alter their morphology and increase engulfment of β amyloid, a protein thought to be involved (if not initiate) a series of neurotoxic events in AD (Iaccarino et al., 2016).

To achieve the same effects non-invasively, we harnessed the neural circuits’ natural tendency to respond to sensory stimuli. While extensive prior work has shown that flickering lights and sound stimuli drive neural activity at specific frequencies in sensory cortices, we have found such flickering sensory stimuli can also drive specific frequencies of neural activity in deeper brain structures, including the hippocampus, albeit with more modulatory effects (Iaccarino et al., 2016). Using this approach, we demonstrated that flickering stimuli within gamma frequencies, 40 Hz in particular, drives gamma frequency neural activity in hippocampus and recruits microglia to engulf pathogenic proteins in mouse models of AD. Existing methods for stimulating neural circuits with temporal precision are either invasive (e.g., DBS), or only access superficial brain structures [e.g., transcranial electrical stimulation (tES)]. Thus, the demonstration that flickering sensory stimuli drives rhythmic neural activity in deep structures reveals a new, and potentially powerful non-invasive tool with which to modulate and/or manipulate neural activity. Moreover, the discovery that stimulating neural activity at specific frequencies can recruit immune cells in the brain to reduce molecular and immune cells may afford a new potential use of neural stimulation to treat certain forms of neuro-psychiatric disease.

### Novel Databases for DBS and Neuroimaging Stereotactic Techniques

#### CranialCloud

The use of complex, heterogeneous, multi-level and -dimensional data is -and will increasingly be – crucial to realizing the potential of a number of neurotechnologies and their use in clinical practice (DiEuliis and Giordano, 2016). Rapidly advancing technology in healthcare informatics is providing increasing opportunities for innovative strategies in both the clinical management of patients, and the advancement of research. As informatic technology is more widely adopted into clinical practice, increasing opportunities – and challenges – arise in the collection, storage, analysis, interpretation, and use of ever larger and more multi-dimensional data resources. Many laboratories throughout the world are using varied imaging and neurophysiologic techniques to address research questions and the translation of positive experimental findings into clinically viable methods. Inter-institutional collaboration (on both intra- and inter-national scales) requires standardization of the data collection process and effective and efficient means of data sharing. However, much of the collected data is stored locally, and sharing capabilities are either expensive, inefficient, or unavailable. Therefore, it will be important to develop improved methods for facile exchange of information between large datasets and patient cohorts.

Cranial Cloud engages this opportunity by providing a platform to standardize data collection and storage (D’haese et al., 2015). This program is equipped with tools to process, analyze and share data in a de-identified manner. Similar in design to the *DropBox* model, the Cranial Cloud system is capable of integrating information with currently available and widely used electronic medical record systems (e.g., EPIC). Clinical assessments and anatomical/imaging data can be collected in a standardized method in order optimize compatibility with various atlases and programs. The overall goal is to maintain and advance normalized, consistent and efficient data collection, sharing and analysis soas to both reduce (if not eliminate) constraints imposed by existing systems, and to avoid the time and economic costs of duplicative analyses. Questions remain about the funding source of such an endeavor, but it will likely require collaboration between academic, corporate and government institutions. Concerns about data sharing with third parties will be a challenge for ensuing technologies.

#### Blackfynn

National Institutes of Health policy states that data should be widely and freely available, while still maintaining privacy and confidentiality^1^. In keeping with this policy, the Blackfynn Scientific Platform functions to standardize data collection in DBS patients, and to make these data more accessible in accordance with existing regulations for data sharing^2^. As well, this platform can maintain data that would otherwise be lost due to cessation of funding. Blackfynn aims to develop a strong business model to sustain the database independently of short-term funding. This database will enable improved workflow and medical record integration that will appeal to clinicians and researchers. A major feature of the program is its web-based, high performance computing capability, which allows users to maintain possession of their data while simultaneously using the platform for data analysis.

One example of such successful data collection, storage and sharing is the Parkinson’s Progression Markers Initiative (PPMI), which facilitates rapid exploration and data-driven analysis, rather than exclusively utilizing a hypothesis-driven approach. This database can also be combined with other backgrounds and datasets to examine biomarker overlap and/or to integrate genetic or physiologic data, and can facilitate cooperation and collaboration between the academic and corporate sectors, and can positively impact and be implemented by a variety of stakeholders, including foundations and advocacy groups. These collaborations are – and will be ever more – essential given the resource demands for the sustenance and iterative expansion and improvement of such a large database.

#### Surgical Information Sciences

The neurosurgical implantation of DBS systems requires a high degree of accuracy in placing electrodes at the identified target sites, and clinical outcomes greatly depend upon the precise localization of special leads and contacts (Ellis et al., 2008). For example, suboptimal STN electrode placement has been associated with reduced clinical efficacy and the occurrence of adverse effects. At present, all candidates for DBS implantation surgery undergo 1.5 (i.e., structural) and/or 3 Tesla (T; functional) magnetic resonance imaging (MRI) as a component of the presurgical clinical workup. With the widespread availability of MRI devices for clinical use, direct targeting (i.e., targeting that is based on visually identifying the intended sites of electrode placement) has become more feasible. However, standard clinical MRI protocols are typically associated with low resolution, inadequate image contrast and relatively low signal to noise ratio (SNR), which make accurately identifying the intended anatomical targets difficult. As a result, it can be challenging to identify the exact borders of the intended targets, and differentiate DBS targets from adjacent anatomical structures.

**FIGURE 10:**
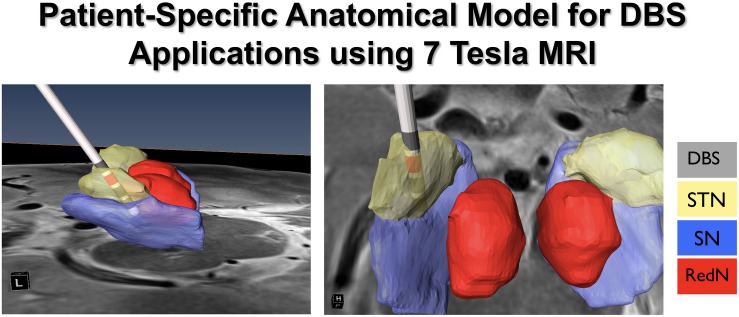
Patient specific modeling for DBS.

Ultra-high field (UHF) MR acquires images using a generated magnetic field that is equal to or greater than 7T (Abosch et al., 2010). 7T MRI has proven to provide structural images of the human brain with rich informational content, higher contrast and resolution, and with the potential for use in clinical applications (Duchin et al., 2012, 2018; Gunalan et al., 2017). The enhanced images acquired using 7T systems improve the ability to visualize subcortical structures, and to create patient-specific 3D anatomical models. This is important given that there is significant variability across individuals in the location, volume, length, depth, and width of a number of neuroanatomical structures (e.g., the STN). The use of patient-specific 3D anatomical models using pre-operatively acquired 7T MRI data can be combined with post-operative imaging (CT or MRI) to create computer-generated depictions of DBS electrode position and contacts (Duchin et al., 2018) (Figure 10). Additionally, the use of diffusion weighted images and structural connectivity-based parcelation protocols can enable depiction of STN connections to the motor, limbic, and associative cortical areas, which can be used to map the individual subdivisions of the nucleus (Plantinga et al., 2018). These new capabilities suggest that the use of 7T MR imaging may facilitate individualized and highly specific planning of DBS implantation of the STN, as well as other DBS targets (Patriat et al., 2018; Shamir et al., 2019). Our ongoing work is focused upon exploring these capabilities in further detail.

### Neuroethical, Legal, and Social Issues (NELSI) in DBS

#### Continued Access to Investigational Brain Implants and Care

At the most fundamental level, the aim of development and use of brain implants has been to enable successful intervention against treatment-resistant neurological and psychiatric disorders. Although the underlying rationale and goals of invasive neuromodulation were and remain benevolent, implantation of these devices exposes patients to both the inherent risks of neurosurgery and to the burden and risks incurred through the use of novel technology (Giordano, 2015a). There is growing discourse about if and how DBS systems should be removed (i.e., explanted) if satisfactory or durable clinical outcomes are not achieved, or if deleterious/adverse effects are incurred. Most trials include plans for explantation for those patients who do not receive benefit or who may have adverse effects, but generally, the cost of explantation surgery is not covered. This is not unique or surprising, given that other incurred costs (e.g., unscheduled visits to the clinical specialists, battery replacement, device repairs, salvage procedures due to infection) may – and often – also are not covered (Rossi et al., 2017).

To date, such issues have been addressed on a case-by-case basis. In the United States there is no legal obligation to provide continued access to DBS devices or resources. Yet, given that the majority of DBS implantation is undertaken as either a large scale clinical trial, or as a component of investigator initiated research (IIR) studies, the constructs of the Declaration of Helsinki could be utilized to support and reinforce continuity of care under as a component obligation of protecting the best interests of the patient/subject – both during and as a consequence of the research study – as constituent to the conduct of responsible research (Giordano, 2015b, 2016). The strength of the moral obligation to provide continued provision of, and access to care depends on the vulnerability of patients, the burden placed on them by the research protocol (e.g., lack of treatment options, and imperatives to advance these technologies), and the feasibility of providing continued clinical care without unnecessarily impairing or impeding ongoing or future research.

Indeed, costs of establishing, and supporting continued care could substantially constrain certain sponsors’ ability to pursue research. In turn, such constraints could negatively impact both a sponsor’s engagement in and support of further research, and the potential benefits that such research could provide to the extant and future recipient patient populations at-large. Recent requests for proposals (RFPs) issued via the National Institutes of Health’s programs within the US Brain Research through Advancing Innovative Neurotechnologies (BRAIN) Initiative have mandated that researchers describe the plan for the patient at the end of the study period, and the ethical principles supporting an extended (albeit) limited duty of care beyond the research protocol. More specific language is being considered for incorporation into current and future RFPs. But any meaningful discussion of ethics must acknowledge and address economic issues, and so consideration of continued care as a constituent of responsible research must be inclusive of cots and coverage of provision and access, time period of extended care, availability of treatment options and alternatives, development of novel treatments, and the systems in place and operational in order to accommodate the fiscal burdens of such needs and demands.

#### Toward Ethical Integration in the Development and Use of DBS

Perhaps one of the more provocative, if not contentious ethico-legal questions is whether DBS “changes” personality, the “self” and in these ways, impairs patient individual autonomy (Jotterand and Giordano, 2011; Giordano, 2015b). As well, there are concerns about patients’ and families’ psychological effects and reactions to living with the device; the effect of DBS on patients’ social relationships; and to broader influence that DBS – and other neuromodulatory technologies – will incur upon and within society and culture. The question of whether stimulating brains is a form of “mind control” and “creating new selves” is gaining prominence as a focus of discourse, both within medicine and the social sciences, as these technologies and techniques are being both considered for the treatment of a broader palette of neurological and psychiatric disorders, and viewed toward the potential to modify cognition, emotion and behavior in “cosmetic,” socio-politically and/or military contexts and ways. Looking – and moving – ahead, qualitative and behavioral studies in natural environments will likely be increasingly necessary to more fully elucidate the ways that DBS (and other neuromodulatory technologies) can, should, or should not be developed and used to mitigate or prevent the effects of disease and injury, and/or improve the quality of life.

Moreover, defining “advancement” and “improvement” to establish the groundwork for ongoing research, development and use-in-practice will be essential to a prudent approach to DBS in this era of personalized medicine, global health initiatives, healthcare and technological inequalities, and the expansion of multi-national research and medical tourism opportunities. In this light, such discourse must be international and sensitive, if not responsive, to differing cultural perspectives, needs, philosophies, values, and capabilities (Stein and Giordano, 2015; Giordano, 2018). We have previously advocated for “no new neuroscience without neuroethics” and “no neuroethics without neuroscience” (Giordano and Shook, 2015), as ethico-legal and social discourse and decisions must be based upon and proceed from the realistic capabilities conferred by the science and technology. Here, we widen our invocation to appeal for a multi-cultural lens, discourse and engagement soas to fortify scientific and technological developments with a fuller depiction and consideration of the socio-cultural contexts and realties that may shape and be shaped by the use of DBS on the world stage.

## Summary and Conclusion

**FIGURE 11:**
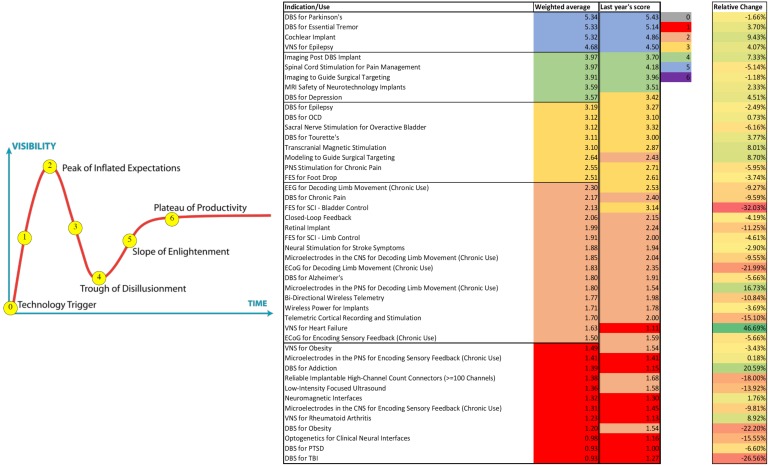
Perspectives and Attitudes Toward DBS. An anonymous 40 question poll was sent online to assess participants’ perspectives and attitudes toward the current and near-term future developments and applications in the DBS field. Sixty six participants responded.

The field of neuromodulation continues to evolve and ongoing research will shape the scientific landscape. This manuscript represents the views of the participants of the think tank and does not consider all the literature on the topics discussed. As in prior years’ meetings, an anonymous 40 question poll was sent online to evaluate participants’ perspectives and attitudes toward the current and near-term future developments and applications in neuromodulation. Sixty six participants responded. Figure 11 summarizes these responses and compares them to last year’s responses. It is notable that some applications moved to the trough of disillusionment (e.g., DBS for depression), others moved to the peak of inflated expectations (e.g., Vagus nerve stimulator for heart failure), and others remained on the slope of enlightenment (DBS for Parkinson’s, DBS for essential tremor, cochlear implants, and VNS for epilepsy). Consistently, the use of DBS for movement indications has reached the plateau of productivity and among most Think Tank participants there was cautious optimism regarding the use of larger network based modulation. There was also cautious optimism for advancing neurophysiological and anatomical signals to improve neuromodulation for several neuropsychiatric conditions, non-neurological indications. There was clear optimism for the development of novel technologies.

## Ethics Statement

Individual studies were approved by the local Institutional Review Board of participating institutions in this technical report and written informed consent was obtained from all participants.

## Author Contributions

AR-Z, JG, EB, VG, AG, PS, SS, CM, MF, JV, VV-M, UA, LA, HB-S, HM, NP, AHG, AS, MC, GL-M, MR, MAR, LC-Z, P-FD, WC, RG, HC, AK, KB, JW, WD, NH, WD, KF, and MO fulfilled the authorship criteria by substantial contributions to the conception of the work, providing data for the work, revisiting it critically for important intellectual content, approving the final version, and agreeing to be accountable for all aspects of the work in ensuring that questions related to the accuracy or integrity of any part of the work are appropriately investigated and resolved.

## Conflict of Interest Statement

The authors declare that the research was conducted in the absence of any commercial or financial relationships that could be construed as a potential conflict of interest. The reviewer VV-V declared a past co-authorship with one of the authors MO to the handling Editor.

## Footnotes

1 https://grants.nih.gov/grants/policy/data_sharing

2 https://www.blackfynn.com
